# Evaluating the effects of a multicomponent support service for people recently diagnosed with dementia and their carers: A qualitative study

**DOI:** 10.1111/hex.13767

**Published:** 2023-04-21

**Authors:** Jonathan Ling, Karen McCabe, Ann Crosland, Laura Kane, Judith Eberhardt

**Affiliations:** ^1^ Faculty of Health Sciences and Wellbeing University of Sunderland Sunderland UK; ^2^ Department of Psychology, School of Social Sciences, Humanities and Law Teesside University Middlesbrough UK

**Keywords:** dementia care, holistic services, peer support, people living with dementia

## Abstract

**Introduction:**

Although prior research has provided an understanding of the needs of people living with dementia (PLWD) and their carers, less is known about how tailored multicomponent interventions impact their lives. This study explored the effect of providing ongoing support to people who had been recently diagnosed with dementia and to their carers.

**Methods:**

We conducted interviews with a convenience sample of key stakeholders: 11 interviews with people who had dementia and their familial carers (*n* = 14) and six interviews with staff and other practitioners involved with the service (*n* = 13). Inductive thematic analysis was performed on the data.

**Results:**

Four themes were developed: the service as a source of respite, peer support, activities as facilitators of emotional wellbeing, and social support. The service was well‐respected, credible, and trusted and was highly valued by practitioners, clients, and carers. It had a clear role in supporting PLWD and their carers. Peer support provided through the service contributed to greatly reducing self‐reported carer burden.

**Conclusion:**

Recommendations arising from this study include offering holistic services to PLWD and their carers, developing activities for men, raising awareness of services among practitioners working with PLWD, and improving partnerships between services and agencies working with older people.

**Patient or Public Contribution:**

Service users were consulted on the themes generated from the data and were asked to provide feedback to help guide the interpretation of the data and ensure this reflected their views and experiences.

## 1 INTRODUCTION

In the United Kingdom, one in three people aged 65 or over will develop a form of dementia.^1^ Although symptoms and progression of dementia vary widely between individuals, typically, as the disease progresses, people need more assistance with their activities of daily living and require support to enhance their functional capacity.^2^ Dementia exerts a significant emotional impact on families, and a substantial economic burden on carers and society more generally. For example, the cost of dementia to the United Kingdom was estimated in 2019 to be £34.7 billion/year, by 2040 the total cost of dementia in the United Kingdom is estimated to increase by 172%, equalling approximately £94.1 billion.^3^

Improving support for people with dementia to live independently and to support their carers is important to reduce the substantial associated social and economic burdens of dementia.^4^ Unpaid care, typically provided by familial caregivers, saves the United Kingdom £13.9 billion annually^3^; however, three out of five familial caregivers reported their caring role, including a lack of support from services, financial losses, and coping with the symptoms of dementia, adversely affected their health and wellbeing.^4^ Informal carers continue to be relatively invisible in policy, practice and research.^3^ Many people are happier if they can remain in their own homes for as long as possible^5^ and providing health and social care support to families affected by dementia reduces avoidable hospital admissions and institutionalisation.^6^ That said, little work has focused on how these support services should operate, particularly related to the needs of those who are using them.^7^

While family caregivers of people living with dementia (PLWD) have reported a heightened sense of fulfilment,^6^ providing such care has a negative impact on physical and psychological wellbeing,^8^ including a heightened risk of cardiovascular disease, cognitive decline and mood disorders,^9^, ^10^ morbidity^11^ and suicidal ideations.^12^ High levels of caregiver burden are associated with an increased risk of early institutionalisation for PLWD.^10^ An Alzheimer's Society report^13^ examining provisions for PLWD and their family caregivers highlighted 65% of family caregivers felt they were not provided with enough support to care for their relative living with dementia, 61% reported their caregiving role had negatively impacted their health, 18% felt depressed, 35% reported feeling stressed and 27% reported feeling cut off from society. These statistics are of significant concern, considering that 87% of PLWD are cared for by a family member.

Carers' feelings of social isolation and loneliness^6^ echo those of PLWD who feel the loss of social connectedness to their family and others in their lives.^4^ Recent research has evidenced a significant increase in PLWD and their caregivers' levels of stress, anxiety, social isolation and loneliness^14^, ^15^ due to the COVID‐19 pandemic, which led to closures and decreases in social support services and respite care.^15^ Caregiver burden has also increased during this time,^15^ which occurs when the caregiver experiences a combination of role captivity, fatigue, burnout, adverse life experiences and a diminished relationship between themselves and their relative.^16^ It is thus paramount to ensure access to and equity of support services postpandemic.

Daycare centres are one of the most common support services, providing essential respite to family carers and social support to PLWD, thereby improving the wellbeing of both parties^7^ and helping reduce behavioural and psychological symptoms of dementia, depression, inactivity, and improved self‐esteem in PLWD. Caregivers also report an increased sense of competence in their role and decreased levels of burden, resulting in an overall delay in the institutionalisation of PLWD.^17^, ^18^

Daycare and community centres give PLWD and their caregivers access to social support and peer support for both; research has evidenced the efficacy and value of peer support in meeting the unmet needs of both populations.^19^, ^20^ Peer support can promote adaptive coping, reduce uncertainty, and normalise the lived experience.^21^ A systematic review examining the efficacy of peer support for family caregivers of PLWD on wellbeing, demonstrated the benefits of a two‐tier model of support, including peer support and supplementary professional support to improve caregiver psychosocial wellbeing.^22^ However, further research is required to understand the most beneficial ways of supporting PLWD and their caregivers.

Multicomponent interventions combine a minimum of two theoretically different approaches, such as peer support, training and educational support, and psychological support.^23^ Such interventions benefit PLWD and their family caregivers, especially those providing access to peers who have shared lived experiences.^24^ Thus, multicomponent interventions take a holistic approach, addressing individuals' emotional, social and educational needs.^23^ Previous research has shown the importance of such approaches in supporting PLWD and their carers, especially approaches underpinned by the adaptation‐coping model.^17^, ^18^, ^23^, ^25^ The adaptation‐coping model^26^ proposes seven adaptive tasks for PLWD, which are associated with quality of life and wellbeing. These tasks include dealing with one's diagnosis of dementia, maintaining emotional balance, developing and maintaining positive social relationships and developing positive relationships with relevant health and social care professionals. According to the adaptation‐coping model, successful or unsuccessful adaptation affects the quality of life of PLWD.^27^ Unsuccessful adaptation has been associated with more behavioural and psychological symptoms of dementia.^27^ Attendance at meeting centre support programmes has been associated with improved quality of life of PLWD and decreased behavioural and psychological symptoms of dementia.^25^ Day centres enable access to peers and professionals for PLWD and their familial carers and therefore promote adaptive coping and improve the wellbeing of both parties.^23^, ^24^

Although prior work has helped to establish an understanding of the needs of carers of PLWD, less is known about how interventions specifically tailored for them has an influence on their lives. The present study explored the effect of providing ongoing support to people recently diagnosed with dementia and their carers. The aim of this support was to help people to live well with dementia for as long as possible and enable them to have a full and active life in the community.

This work examined the following research questions:

1. What are the perceived needs of people recently diagnosed with dementia and their carers when a diagnosis is made and how does the service meet those needs?
2. What are the most appropriate measures of success from the perspectives of people recently diagnosed with dementia, their carers, and service providers?
3. How effective is the service in meeting the needs of people recently diagnosed with dementia and their carers?

## 2 METHODS

### 2.1 Design

The current study used a qualitative approach, employing inductive thematic analysis^28^ as well as the constant comparative method,^29^ a method which, although originating from grounded theory, can be applied outside of grounded theory.^30^ Our approach was driven by pragmatism, focusing on the potential consequences of any findings emerging from the data.^31^

The setting was a multicomponent service in North East England provided by a charitable organisation, dedicated to PLWD and their informal carers. Its expertise centred around an extended support, information and advice service to carers accessing the service, including training on dementia. The service also offered leisure and learning activities tailored to the needs of their clients (e.g., coffee mornings, arts and crafts, walks, and men‐friendly activities such as boccia and reminiscence sporting memories).

### 2.2 Participants

Client and carer participants who took part in this study had recently received a diagnosis of dementia and/or been involved with a dementia support service for varying periods of time from around 9 months to over 2 years. In addition to living with dementia, many client participants were also living with other physical and/or psychological conditions and therefore had complex needs. All PLWD were all aged between 60 and 79 years. Participants were referred to the service through a variety of routes. These included referrals from hospital discharge, primary care, occupational health, Age UK, and self‐referrals (including referrals made by carers and family members).

Data collection took place via 17 semi‐structured, qualitative, face‐to‐face or telephone interviews with 27 key stakeholders of the service who were all based in the North East of England. We conducted 11 interviews with people who had dementia and their familial carers (*n* = 14) and six interviews with staff and other practitioners involved with the service (*n* = 13).

### 2.3 Recruitment

Practitioner stakeholders were recruited via purposive sampling. They were initially approached to participate via email. Written information about the study, confidentiality, informed consent, and what participation would involve was provided and a mutually convenient date and venue for the interview to take place was arranged.

PLWD and carer stakeholders were recruited via convenience sampling. All client and carer stakeholders were initially approached to participate by staff at a service for PLWD and their carers. All PLWD and carer participants were provided with written information about the study, confidentiality, informed consent, and what participation would involve in advance of the interview.

### 2.4 Data collection and analysis

All interviews were conducted by the same researcher (K. M.). A constant comparative approach^29^ was used within data collection and analysis whereby issues raised in earlier interviews informed the direction of later interviews. For example, several interviewees talked about the service as a source of respite, which resulted in modification of the interview schedule to include further questions to probe this line of enquiry in more depth in subsequent interviews. All interviews were digitally audio‐recorded and transcribed verbatim, anonymising all personal and professionally identifiable information.

Data analysis followed a six‐step thematic approach.^28^ All data analysis was carried out on the transcripts by hand by the researcher who conducted the interviews, and themes were then checked against the data and verified by the first author. Furthermore, member checking was employed to ensure trustworthiness; service users were consulted on the themes generated from the data and were asked to provide feedback to help guide the interpretation of the data and ensure this reflected their views and experiences. The study was reported according to the Standards for Reporting Qualitative Research.^32^

## 3 RESULTS

The thematic analysis led to the development of four themes from the data: (i) the service as a source of respite; (ii) activities as facilitators of emotional wellbeing; (iii) peer support; and (iv) social support.

### 3.1 The service as a source of respite

On accessing the service, clients and carer participants were overcome by the care and attention they felt was afforded to them and/or their loved ones, describing their initial contact with the service as ‘wonderful’, ‘welcoming’ and as providing them with a ‘huge sense of relief and comfort’.

*My wife went down [to the centre] and started to change. She started to get more confidence. We felt as though the service had put an umbrella over us*. (Carer 5)

Before accessing the service, many clients and carers stated that they felt anxious, stressed and frightened. Some participants reported suffering from depression. Many participants felt that, throughout the diagnosis period and after, they had drifted from hospital department to department and organisation to organisation, sometimes with little follow‐up or satisfactory resolution to their problems, whether clinical, medication, practical (e.g., mobility aids) or emotional. During this time, participants stated that they constantly had to re‐tell ‘their story’ to differing people and at times described feeling dehumanised—defined by the diagnosis of dementia.

The person‐ and family‐centred service provided was greatly valued by clients and carers. They stated that they felt they were perceived and treated by the service staff as ‘a person; a couple; a family’.

*The sense of relief that I personally experienced when I got in touch with the service was just immense. I cannot begin to put into words how it felt to walk in to somewhere where they understood. It was the first time that I actually felt that they weren't seeing dementia, they were seeing a person*. (Carer 1)

This was supported by clients' perceptions. Processes and problems encountered after receiving a dementia diagnosis were described as feeling considerably smoother once the service had become involved and there was great trust among clients and carers in the service and the staff. Participants stated that this had developed from the ability of the staff to provide help and/or support—whether emotional or practical. Often staff would exceed expectations and would provide rapid information or support, seemingly effortlessly and typically in ‘super‐quick time’.

*To me, they've got that helping thing. […] That, oh, right ‐ if I want something, I ask them—and it happens*. (Client 9)

When describing their perceptions of the overall impact of the service, participants described the service as the highlight of their day or week; a place they looked forward to attending and a service that provided everything they needed. Some described their involvement with the service as the best thing they had ever done, directly improving their mood and giving them increased self‐confidence. Some clients related how they were now described by other family members as being a different, more sociable and happier person, and that the staff and the wider ethos of the service made them feel special.

*This place is the best thing that ever happened to me. I mean, I come along any day, like. Any time, I sit down and have a bit talk and a cup of tea or coffee. You know, I was made welcome…. You come here, it's just like a family home. You can sit and just talk to people, let your hair down, carry on*. (Client 8)

Clients described the importance to them of knowing that when they came to the service they would feel at ease, being with ‘people just like me’ (Client 10) in a welcoming, supportive, safe, non‐judgmental environment. The service was considered to be more receptive than generic community activities. Many clients and carers felt that there remained much stigma and lack of awareness of dementia within the wider population and described their wariness of being noticeable or visible within the community. While some clients did access generic community activities, for others these were considered to be frightening, too busy, too noisy and too ‘fast’.

*Some people can be ignorant as well. And they just expect you to get on with it and life is not like that now. You know, you're [following] your guidance [from healthcare professionals on managing the condition] and you need to know. Be told what you've got to do. But if you go to places like that, they just—they're not really interested*. (Client 10)

### 3.2 Activities as facilitators of emotional wellbeing

The service provided a range of activities including curling, crafts, computers, a men's group, and a carers' peer support group/coffee morning. The activities provided within the service were largely well‐received although personal preferences differed. People accessed the service to take part in the activities they enjoyed. Some client and carer participants stated that they did not access any other services, mainly due to the trust they had in the service. Men attending the service as carers or clients were very engaged, and motivated to take part and reported greatly enjoying accessing activities.

A trip to a local outdoor museum was unanimously perceived as memorable and special—within this trip, the visit to a 1940/1950s cottage and singing along to ‘old songs’, accompanied by a piano, were specifically recalled. This was described by all participants as a very positive and emotional experience. Participants described how everybody, most notably those with dementia, sang along to the songs.

*When we were up at [the museum] – the end of the afternoon, somebody came in and started playing the piano and playing all the old songs. And [Name] was just sitting there, singing. And the amazing thing is she knows the words*. (Carer 6)

Several carer participants stated that they wanted to or were already attending the support service on their own when their loved one attended other activities or services. Carers felt that this was beneficial both because of the support they felt they received from attending the service and because they wanted to share their experiences and knowledge, to ‘give something back’ (Carer 2) to the service and other carers.

### 3.3 Peer support

The service was considered by client and carer participants to be a stable source of support—a constant, and familiar entity. This mainly took the form of peer support. Many clients and carers referred to staff and fellow clients as family or extended family, and spoke of making new friends or of renewing old acquaintances. One client described the service as being somewhere they could confidently talk freely anytime. Clients and carers considered the support and information that they drew from others when attending the service, or from friendships made through it, to be invaluable.

Among carers, peer support was considered especially important and the activities that were organised for carers, most notably the coffee morning, were highlighted as particularly important. Carers stated that being able to speak with other carers, share experiences, find out that one was not alone in experiencing them and discuss ways of dealing with them was enormously helpful and supportive for them. Having the opportunity to just ‘offload’ to others who understood and may have had similar experiences was also highlighted as beneficial.

Clients, on the other hand, valued interaction with people at differing stages of dementia in an atmosphere and location where everyone was comfortable and accepted for being themselves. Clients, who were all in the early stages of dementia, stated that they were not daunted by their own future when interacting with those who were experiencing later‐stage progression; indeed, they reported that they and their carers and families were helped by getting an insight into the issues to expect with dementia progression.

Carers and clients valued sharing information about other services, support and activities that were available and particularly valued others' recommendations.

*The cross‐fertilisation that goes on between carers, as a result of the service is brilliant. And, in a sense, you know, in some ways that's better. Because if another carer is suggesting that something is working for their partner or, you know, mother or father, you're more likely to actually think, oh well, you know, yeah, maybe that would be worth trying*. (Carer 1)

### 3.4 Social support

Social support emerged as a further theme from the interviews, with many clients and carers stating that if they had not been involved with the service, they would have been more socially isolated, unhappy and withdrawn. Responses from some participants were more emotive—they stated that they could not express how they would have coped without the support service; some had previously experienced suicidal thoughts and one carer emphatically stated that the service had ‘saved my life’.

Additionally, many participants reported that without the support of the service they would not have been able to cope as well with financial issues, or with accessing practical or mobility aids—issues which impact physical and mental wellbeing and overall quality of life.

All clients and carers stated that aside from specific clinical/medical issues or when emergency care was needed, their first point of contact for support or problem resolution would be the service. Many specifically stated that they would contact the service as their first response before contacting their general practitioner (GP) or other agencies. Some carers recounted how involvement with the service had already reduced their own visits to the GP for anxiety, and stress or depression symptoms had been alleviated, and that they generally felt better and considered themselves more able to cope since becoming involved with the service.

*I don't know—they're just there for you. If you need them, they're there. You know, you've just got to ask and they're there for you*. (Carer 3)

## 4 DISCUSSION

The present study explored the effect of providing ongoing support to people who had been recently diagnosed with dementia as well as to their carers. Generic themes that emerged across all stakeholder groups identified that the service was well‐respected, credible and trusted and was highly valued by practitioners, clients, and carers alike. Findings from this study indicate that providing a service for PLWD and their carers had a clear role in support for them and their families.

The findings of our study align with the findings of previous quantitative research,^18^, ^19^, ^25^, ^26^ supporting the potential benefits of multicomponent interventions and day centres. The qualitative nature of this study complements previous quantitative research by providing a deeper understanding of which components of support services are preferred for PLWD and their carers. Further knowledge about which individual components are of most benefit within the larger system of support is needed.^24^

Meeting the support needs of both PLWD and family carers is pivotal.^6^, ^14^ Day centres for PLWD and their carers provide much‐needed respite and access to social support from peers and professionals,^7^ and increase adaptive coping.^25^ The positive impact of a two‐tier model of support (peer and professional), implemented in this study, is supported by previous literature,^22^ highlighting the importance of providing PLWD and their familial caregivers with access to informational support and knowledge and emotional support obtained through shared lived experience.^20^ Furthermore, reducing the caregiver burden is important to both the health and wellbeing of PLWD and their carers,^16^ which has increased significantly due to the COVID‐19 pandemic and the closure of day centres and other support services.^14^, ^15^

Based on our findings we propose the following areas for further investigation.

### 4.1 Holistic services for PLWD and their carers

The service examined in the present study was highly valued by clients and practitioners alike. Similarly, the client support provided by the service that enabled carers to access support or activities was highlighted as invaluable by participants. Client, carer and practitioner participants reported that involvement with the service had a positive impact upon clients' and carers' general, emotional and mental wellbeing, crisis management and dementia progression. While such service provision is difficult to resource, both financially and physically, the service provides comprehensive support for clients and carers elsewhere. Our findings are congruent with findings that both PLWD and their familial carers benefitted from accessing a service within the community which offered educational, informational and psychosocial support.^17^, ^18^ Support for such provisions, although more expensive short‐term, may reduce long‐term costs through improving PLWDs' quality of life and delays in institutionalisation.^7^ This needs to be further explored.

### 4.2 Activities for men

The service in the present study operated a specific men's group as well as several other activities which, while not exclusively for men, were well attended by male clients and carers, such as a film club. Earlier work^33^ acknowledges that men are difficult to engage in positive health‐related behaviour change. We recommend that, while not ignoring the needs of female clients or couples, future research should examine the usefulness of similar support services offering activities that men engage with to enable the delivery of additional health messages through these.

### 4.3 Awareness raising of services among practitioners working with PLWD

In this study, all stakeholder participants recounted examples of practitioners they had encountered who were unaware of such services. Promoting services such as the one examined in the present study among relevant professionals is important to provide support for PLWD and their familial carers. Services could identify those professional roles most likely to have contact with people who would potentially benefit from them. Once identified, ascertaining the most appropriate methods of engagement and communication for them should be considered. Support services could also raise awareness of their offer by linking with existing local networks or websites that promote wellbeing.

Understanding the potential unknown and unmet needs of different groups diagnosed with dementia should inform any future research with services for PLWD and their carers.

### 4.4 Partnerships between services and agencies working with older people

Some duplication of services was reported in our study. While access to a wide range of services provided by different organisations offered improved choice and opportunities to select support and activities most appropriate to individual needs, duplication (most notably giving and re‐giving information) and lack of coordination across services was found to be frustrating. Crucially, this study established that once accessed, the support service provided a ‘one‐stop‐shop’ approach to support and care. However, some duplication existed among both dementia‐specific and generic services, especially those providing support for older people—including local housing providers and charities. Closer partnership working and networking would result in a greater level of awareness of service provision across organisations to ensure clients and carers are at the centre of care, and to reduce duplication. Future work needs to examine how existing services could be modified to facilitate partnership working. This could include developing a more comprehensive library of resources for other available services and utilising PLWD and carers more in service delivery.

### 4.5 Strengths and limitations of this study

While this was a small‐scale qualitative study, data saturation was reached within this sample size and the timescale of the study. The use of the constant comparative method^29^ enabled the verification of themes identified, allowing these issues to be explored in depth.

Group interviews for some practitioner stakeholders enabled us to gain wider access to practitioners. This was a pragmatic decision taken by the research team to maximise participation; however, this may have increased normative responding from the group ‐ in other words, reduced the likelihood of people voicing views that were not aligned with those of the rest of the group. However, we used member checking with individual interviewees to ensure the trustworthiness of the analysis and thereby help address this issue.

Access to the client and carer stakeholders within this study was brokered via service staff. Accessing participants in this way was necessary to ensure clients and carers were physically and psychologically able to participate in this study, were able to provide informed consent and to ensure that participation would not cause clients or carers any stress, discomfort or upset. There was therefore potential for participants with more positive views of the service to be selected by the staff. Data provided by practitioner stakeholders within interviews included references to feedback from their clients; this supported the views of the client and carer participants within this study and confirmed the finding that the service was overall perceived as positive.

Clients were overwhelmingly positive about the service. They were all actively engaging with the service, and therefore this is likely to be reflected in the quotes. Those who did not find it useful would most likely not have returned to the service. Similarly, staff were all highly invested in the programme. However, funding was not secure over the long term, which may have led staff to overly focus on the positives of the service. Future research with similar services should endeavour to follow up with clients who have stopped engaging with the service, as well as former staff, to elicit their views.

Finally, it needs to be highlighted that the PLWD in the present study had been diagnosed fairly recently, and therefore the impact on themselves and their carers may not have fully been realised yet, compared to PLWD who had been living with the condition for a longer time. However, the recency of diagnosis ranged from 9 months to 2 years, which will have allowed clients and carers some time to adjust. Furthermore, due to the progressive nature of dementia, it can be difficult for PLWD and their carers to anticipate the consequences of a diagnosis and therefore to adjust, even after many years.^34^

## 5 CONCLUSION

The support service in the present study was developed to provide support to people who had been recently diagnosed with dementia and their carers. A specific aim of the service was to support people to live well with dementia for as long as possible and to enable them to have a full and active life. The emotional and practical support and opportunities for social interaction provided were highly valued. These factors were reported to positively influence wellbeing and quality of life.

As well as affecting wellbeing, the relationships developed from interaction with staff within the support service facilitated continuing assessment of individual needs which our findings suggest had the potential to improve economic outcomes by reducing the number of contacts with services such as GPs and reducing the likelihood of episodes of crisis. Future efforts to provide support to PLWD and their familial carers should consider implementing, and evaluating, holistic services, activities for men, and awareness‐raising among practitioners working with PLWD and their carers. In particular, a better understanding is needed of what elements of interventions for carers of PLWD best support their needs.

## AUTHOR CONTRIBUTIONS

**Jonathan Ling**: Conceptualisation; methodology; analysis; writing—original draft preparation, writing—reviewing and editing. **Karen McCabe**: Conceptualisation; methodology; investigation; analysis; writing—original draft preparation. **Ann Crosland**: Conceptualisation; methodology; writing—original draft preparation. **Laura Kane**: Writing—original draft preparation; writing—reviewing and editing. **Judith Eberhardt**: Writing—original draft preparation; writing—reviewing and editing.

## CONFLICT OF INTEREST STATEMENT

The authors declare no conflict of interest.

## ETHICS STATEMENT

Ethics approval for this study was granted by the research ethics committee of The University of Sunderland (approval number 285).

## Data Availability

Research data are not shared. The authors elect not to share data. The low sample number and nature of the topic result in possible identifiability of participants, therefore unable to share on ethical grounds.
